# Editorial: Digital approaches in the nutritional prevention and management of chronic diseases

**DOI:** 10.3389/fnut.2023.1341135

**Published:** 2023-12-12

**Authors:** Mara Pereira Guerreiro, Isa Brito Félix, José Camolas

**Affiliations:** ^1^Egas Moniz Center for Interdisciplinary Research (CiiEM), Egas Moniz School of Health and Science, Almada, Portugal; ^2^Centro Hospitalar Universitário Lisboa Norte—EPE, Lisbon, Portugal; ^3^Laboratório de Nutrição, Faculdade de Medicina da Universidade de Lisboa, Lisbon, Portugal; ^4^Instituto de Saúde Ambiental (ISAMB), Faculdade de Medicina da Universidade de Lisboa, Lisbon, Portugal

**Keywords:** chronic diseases, digital, diet, disease prevention, e-health, m-health, nutrition, self-management (self-care)

Chronic diseases are a global epidemic, responsible for the majority of deaths worldwide. Diet and nutrition play an important role in preventing and managing high-burden chronic diseases (1).

Digital technology became popular for supporting the nutrition care process and enhancing dietary self-management through interventions targeting persons at risk or living with chronic disease, health care professionals or both.

Work portrayed in this Research Topic includes the development and validation of a nutrition self-screening tool (IBD-NST) for the management of inflammatory bowel disease by Wall et al. Digitization offers the advantage of automated calculation of measures integral to the tool, lessening the completion workload. Additionally, subsuming the tool in apps and digital platforms can improve access to self-screening and foster integration of care. Another example of digitisation is the work of Verbeke et al., which outlines the psychometric evaluation of a short food frequency questionnaire in Dutch, firstly paper-based and then in a web version. Food frequency questionnaires are amongst the most used dietary assessment instruments, therefore validated digital versions are useful both for research and clinical purposes. Griauzde et al. illustrate how a system for virtual consultations was successfully used to deliver a very low-carbohydrate diabetes prevention programme to US Veterans with prediabetes. Such an approach is promising for improved enrollment and retention, given the low participation of eligible Veterans in the in-person, telephone, or video sessions of the Weight Management Programme MOVE!

Contributions to this Research Topic serve as a glimpse of the extensive range of digital nutritional approaches to prevent and manage chronic diseases. Arguably the most common interventions are those involving self-monitoring of behavioral and health data, through mobile applications (apps), web-based tools and wearable devices.

Apps represent one of the most abundant digital technologies in this field. For example, more than 2,000 potentially suitable apps for diabetes management were identified on the Google Play Store and Apple App Store (2). Among the 120 apps assessed, 77% focused nutritional content (2).

Common features of mobile apps for nutrition management in chronic diseases are food trackers, such as inputting food items through barcode scanning, image capture and automatic image recognition. The latter relies on artificial intelligence techniques and holds significant potential for alleviating the burden associated with self-reporting and human coding. However, the performance of automatic image recognition is currently hampered by difficulties in accurately recognizing the variety of food items in the real world, particularly when considering diverse cultural contexts (3). Food tracking technology enables the generation of nutrition reports, providing information on elements such as calories, macronutrient and micronutrients ingested (3, 4). Often apps allow sharing these reports with health care professionals, thereby assisting in the nutrition care process and serving as a foundation for discussions and counseling of clients.

Quality of mobile apps is a matter of significant concern. To illustrate, Geirhos et al. (2) found that most apps lacked a scientific evidence base. Quality goes beyond the correctness of the information conveyed and demonstrated benefits, encompassing aspects such as usability, privacy and security (5). Digital health assessment frameworks provide a solution to address quality concerns about apps and establish trust among health professionals and the public. These frameworks are intrinsically linked with the emerging field of digital therapeutics, defined as “interventions through a *clinically evaluated, patient-directed* software application intended to improve the process of diagnosing, treating, managing, and/or preventing diseases” (6). Reimbursement of digital therapeutics is a global trend, with Germany standing out as a significant example in Europe. The country has embraced universal reimbursement for prescribed apps, following a national regulatory framework. Two years after the initial approvals, the most commonly utilized apps were those focused on weight reduction (6).

Wearables are portable devices worn on the body capable of recording health or behavioral data, through technologies for, inter alia, identification, sensing, connection, and storage (7). An emerging field of wearable devices in the nutritional prevention and management of chronic diseases is dietary assessment (3, 8). Wearables hold the promise of passive and objectively capturing dietary intake, addressing limitations of diaries and food frequency questionnaires, such as difficulties in self-estimation of portion size and misreporting. These wearables also overcome the reliance on food trackers via mobile apps, which require individuals to actively photograph food intake, a process susceptible to memory lapses and social desirability bias. Dietary assessment wearables being researched currently include sound-based sensors (e.g., ear-mounted microphones to capture chewing sounds and throat microphones to detect swallowing patterns), image-based sensors (e.g., cameras worn around the neck or attached to the chest to classify foods and/or estimate portion sizes based on photographs) and motion-based sensors (e.g., devices attached to the head or neck to detect jaw movements and wrist-worn sensors for the automatic identification of eating).

Additional wearable technologies being tested focus on the passive assessment of specific nutrients (8). This encompasses miniature sensors attached to teeth for detecting salt and alcohol intake and epidermal sensors resembling tattoos capable of identifying diet-related metabolites in perspiration. Such advancements may offer benefits for managing chronic conditions such as hypertension and diabetes.

Several authors have put forward visions of integrated systems (8), in which wearables transfer data to mobile apps and electronic health records, allowing automated just-in-time behavioral interventions and support for health professionals consultations. This vision and the move toward hyper-connected ecosystems is significantly reliant on interoperability (9), which guarantees that systems can exchange information and understand the shared information.

Clearly, the potential of digital behavior change interventions in the nutritional prevention and management of chronic disease goes beyond self-monitoring through apps and wearables, by applying a wider range of behavior change techniques (10). Resorting to standardized sources, such as the Behavior Change Technique Ontology (11), ensures transparency and facilitates replication.

This editorial contextualizes contributions to the Research Topic within the broader landscape of emerging technologies in dietary assessment and global trends. The advancement in these emerging technologies and its mainstream adoption is shaped by various factors, as outlined in the NASSS framework (12). Ultimately these emerging technologies represent opportunities for improving nutritional interventions for chronic disease prevention and management.

## Author contributions

MG: Conceptualization, Writing—original draft. IF: Resources, Writing—review & editing. JC: Writing—review & editing.
